# Injuries of the Posterior Tracheal Wall: Insights from a High-Volume Single-Centre Experience

**DOI:** 10.3390/jcm15010245

**Published:** 2025-12-28

**Authors:** Lavinia Gatteschi, Antonio Burlone, Stefano Bongiolatti, Simone Tombelli, Giovanni Mugnaini, Luca Voltolini, Alessandro Gonfiotti

**Affiliations:** Thoracic Surgery Unit, Careggi University Hospital, 50134 Florence, Italy; antonio.burlone@unifi.it (A.B.); bongiolattis@aou-careggi.toscana.it (S.B.); simone.tombelli@unifi.it (S.T.); giovanni.mugnaini@unifi.it (G.M.); luca.voltolini@unifi.it (L.V.); alessandro.gonfiotti@unifi.it (A.G.)

**Keywords:** tracheal laceration, cervicotomy, hybrid approach, trauma, membranous injuries

## Abstract

**Background**: Major airway injuries, regardless of whether their aetiology is traumatic or iatrogenic, are rare but potentially fatal. In selected cases, surgery plays a key role; however, it has to be performed by highly experienced professionals in emergency settings. **Methods**: We reviewed all surgical procedures involving the trachea which were performed at our institution in the last 5 years (365 procedures). We report here our experiences with major airway injuries, both traumatic and iatrogenic (19 procedures). All patients, including individuals from within our hospital and from other peripheral centres, were treated in an emergency setting within 12 h of correct diagnosis. **Results**: The location and extent of tracheal lesions can be different in every patient. After a proper evaluation with CT scan and bronchoscopy, we approached all our cases of tracheal injuries with a cervicotomy, using, in some selected cases, an endoscopic camera to better visualise lesions that involved the carina. However, in extremely severe cases, such as one we report here, where multiple repair attempts fail and tissue viability is compromised, demolitive surgery by means of posterolateral thoracotomy may represent the only remaining therapeutic option. **Conclusions**: Surgery on tracheal injuries is complex, highly specialised, and time-dependent. In selected cases, it has to be performed quickly by highly qualified professionals after proper evaluation in an emergency setting. Every airway injury differs in its location, extent, aetiology, and clinical presentation, and there is no unanimous consensus on standardising treatment. Only high-volume centres with highly experienced professionals can guarantee correct management of this rare but life-threatening event.

## 1. Introduction

Tracheal lacerations, particularly those involving the posterior wall, represent a rare but potentially life-threatening form of airway injury. These lesions may result from blunt or penetrating trauma, although in contemporary clinical practice the majority are iatrogenic, most frequently following endotracheal intubation, tracheostomy, or endobronchial interventions [1].

Blunt trauma is often caused by high-energy deceleration in motor vehicle accidents or by direct compression of the trachea against the vertebral column, leading to shearing or bursting injuries near the carina. Penetrating injuries, including stab and gunshot wounds, typically affect the cervical trachea and may coexist with vascular or oesophageal lesions. Iatrogenic injuries represent an increasingly prevalent category, most frequently associated with endotracheal intubation, tracheostomy, or endobronchial procedures. The incidence of tracheal laceration following elective intubation is approximately 1 in 20,000–75,000 cases, but this risk increases by up to 15% in emergency or difficult airway scenarios [2].

Risk factors include female sex, advanced age, prolonged or repeated intubation attempts, use of oversized or rigid endotracheal tubes, overinflation of the cuff, and connective tissue fragility.

However, the true incidence of posterior tracheal wall injuries remains difficult to assess. This is largely due to underdiagnosis and delayed presentation, and the associated high prehospital mortality. Indeed, in trauma settings, tracheobronchial injuries occur in approximately 0.5–2% of patients with chest or neck trauma, although up to 80% of severe cases may be fatal before reaching definitive care [2].

The location and extent of tracheal lacerations both vary considerably. Injuries may involve the cervical or thoracic trachea; they may affect the anterior cartilaginous rings or the posterior pars membranacea; and they may be of partial or full thickness. In severe cases, lesions may extend to the carina or main bronchi, or they may coexist with oesophageal tears, leading to tracheoesophageal fistula formation.

Given these complexities, early recognition and prompt management are crucial to improve outcomes in these patients. However, the diagnosis of tracheal injuries is often delayed due to nonspecific symptoms or findings which overlap with other thoracic injuries. High clinical suspicion, bronchoscopic evaluation, and computed tomography (CT) are indispensable for accurate assessment [1].

Despite advances in critical care and attempts to standardise emergency airway surgery like the Thoracic Trauma WSES-AATS guidelines [3], a universally accepted consensus on the optimal management strategy for tracheal injuries is still lacking. Decisions regarding conservative versus surgical intervention depend on the size, location, and depth of the lesion, the presence of associated injuries, and the stability of the patient. Most centres continue to rely on institutional expertise and individualised case assessment to guide treatment.

This article aims to report the five-year experience of our institution in the treatment of posterior tracheal wall injuries, highlighting surgical management and outcomes in these challenging cases.

## 2. Materials and Methods

We present a retrospective, single-centre observational study conducted at our institution. We reviewed all tracheal surgical procedures performed between January 2020 and September 2025: three hundred and sixty-five cases, including both acute injuries and elective surgeries. In this study we exclusively included operations carried out in an emergency setting for post-traumatic or iatrogenic tracheal injuries. Nineteen cases met the criteria. All elective tracheal resection and anastomosis procedures performed for either iatrogenic or idiopathic stenosis were excluded from the study population. We included patients admitted to the emergency department of our institute, patients transported from other hospitals near to the city of Florence, and patients already hospitalised at our centre.

Data were obtained from our institutional operative registers, from anaesthetic charts, and from perioperative and postoperative records. Parameters relating to demographics, the mechanism of the injury, the anatomical location and extent of the lesion, and associated oesophageal/vascular involvement were collected and analysed.

We thus focused on operative details, including the surgical approach and technique, and the use of prosthetic material or muscular flaps. Postoperative variables were also included; these included length of stay in the intensive care unit, length of hospital stay, and the occurrence of any complications (anastomotic dehiscence, infection, stenosis). Further data were collected through clinical evaluation, flexible bronchoscopy, and imaging.

Our Institutional Review Board waived the requirement for formal approval due to the retrospective nature of the study.

## 3. Results

We reviewed our experience of treating acute tracheal injury patients over the past 5 years (2020–2025), and we identified a population of nineteen (19) patients. Fifteen (79%) patients were female and four (21%) were male, with a mean age of sixty-eight years (range 37–85).

Thirteen patients (68%) had a post-intubation tear; four patients (21%), had injuries resulting from a percutaneous tracheostomy attempt; one patient had a penetrating wound stab trauma; and one patient suffered a tracheal injury due to inhalation of a foreign body (dental prosthesis).

Eighteen patients (94%) suffered a lesion of the membranous wall; the patient with the penetrating wound stab trauma had an anterior wall tear only. In all the patients with a post percutaneous tracheostomy injury, both the anterior and membranous wall were injured. Indeed, when this kind of procedure is used, the membranous wall is likely to be injured during the attempt to position the cannula or the dilator through the breach in the anterior wall. This manoeuvre can lead sometimes to an additional rupture of one or more cartilaginous rings, an outcome which may also result from application of excessive strength during cannula positioning attempts [4].

To confirm, the totality of our post tracheotomy cases presented a damaged anterior wall, with one or more tracheal rings found to be collapsed at surgical inspection. From a clinical point of view, in all cases subcutaneous emphysema was present; in ten patients (52%) the membranous wall tear manifested with concomitant dyspnoea and significant limitation of ventilation in those patients who were already sedated; in five cases (26%) the first symptom that occurred was haemoptysis. Every patient underwent an urgent CT scan, and a subsequent fibrobronchoscopy was always performed to confirm the tracheal lesion. In the case of ingestion of a foreign body, an oesophagoscopy was also performed to exclude lesions of the oesophagus.

All the patients underwent surgical repair within 12 h of diagnosis in an emergency setting. In fact, it is widely accepted that early recognition and repair of injuries should be favoured in attempts to prevent the development of airway stenosis [5].

Ten (52%) lesions were located in the thoracic trachea, five (26%) lesions were in the cervical trachea, and four (21%) lesions involved the origin of the right main bronchus. Details are shown in Table 1.

Although injuries differed in their location and their extent, all the patients were approached with cervicotomic access, and in all cases the Angelillo-Mackinlay technique was applied [6]. In three cases we adopted a hybrid surgical approach with a 5 mm 30° endoscopic camera to achieve better visual control of the distal lesion of the membranous wall, especially when the origin of the right main bronchus was involved [7]. Through the endoscopic magnification we proceeded to repair the tracheal and bronchial laceration with a continuous 3-0 absorbable monofilament Polydioxanone (PDS) suture.

Of the membranous lesions, sixteen were repaired with a running suture of 3-0 PDS while two were closed with a 3-0 PDS knotless self-blocking running suture. Closure of the anterior tracheal wall was subsequently achieved with interrupted 2-0 braided absorbable Polyglactin sutures. The average operation time was 103′ (range 20′–180′).

Maintaining oxygenation during surgery is very challenging: all the transtracheal repairs were carried out with on-field intermittent apneic ventilation after partial anterior opening of the pars cartilaginea.

Nine patients (47%) needed a tracheostomy at the end of the procedure; in these cases, a tracheostomic cannula was positioned for a better recovery of the lesion.

A spiral drain (15F) was used in all patients; in the case of the stab wound trauma, a pleural drain (27F) was also added due to post-traumatic pneumothorax.

Thirteen patients (68,4%) were discharged home, and the median length of their stay was 16 days (range 4–38). Of these thirteen patients, twelve (63.1%) had an uneventful recovery from a surgical point of view, while one (5.3%) needed a reintervention for suture dehiscence.

Six (31.6%) patients died in ICU due to complications which occurred after the injury.

### Management of Recurrences

Two cases (10.5%) manifested early recurrences that needed reinterventions.

The first patient manifested issues with invasive ventilation during the second postoperative day, and promptly underwent a bedside bronchoscopy which showed a suture dehiscence. Redo surgery using the previously used cervicotomy approach was performed in an emergency setting, and the membranous lesion was re-sutured with a running suture of absorbable 3-0 PDS. The patient then experienced an uneventful recovery, from a surgical point of view, and was discharged home on the 38th postoperative day, though with other comorbidities.

The second case who presented acute recurrence was accepted in the intensive care unit (ICU) after being transferred from another institute with a diagnosis of trachea-oesophageal laceration which occurred during an emergency orotracheal intubation for respiratory failure due to loss of consciousness. The first surgical repair was attempted with our standard technique. A cervicotomy was performed. First, the oesophageal lesion was sutured with a 3-0 PDS running self-locking knotless suture, and a flap of sternohyoid muscle was positioned between the trachea and the oesophagus. Then, the tracheal lesion was also sutured with a 3-0 PDS running self-locking knotless suture. During the first surgery the tissues appeared extremely breakable, likely due to prolonged exposure to the oesophagus mucosa. A tracheotomic cannula and a 15 Fr spiral drain were positioned at the end of the procedure. The next day, a jejunostomy tube was implanted to ensure proper nutritional support.

Five days after surgery, a limitation to ventilation manifested in ICU, and a bronchoscopic control confirmed dehiscence of both oesophagus and tracheal membranous wall sutures. A second surgery was then performed in an emergency setting.

**Figure 1:**
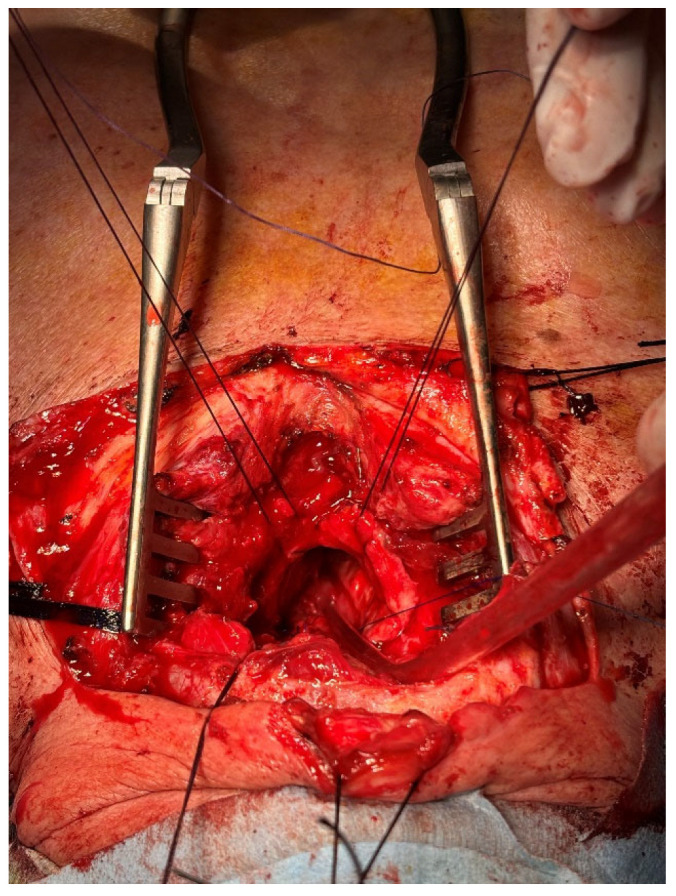
Lesion of the membranous wall with bulking of the oesophagus.

**Figure 2:**
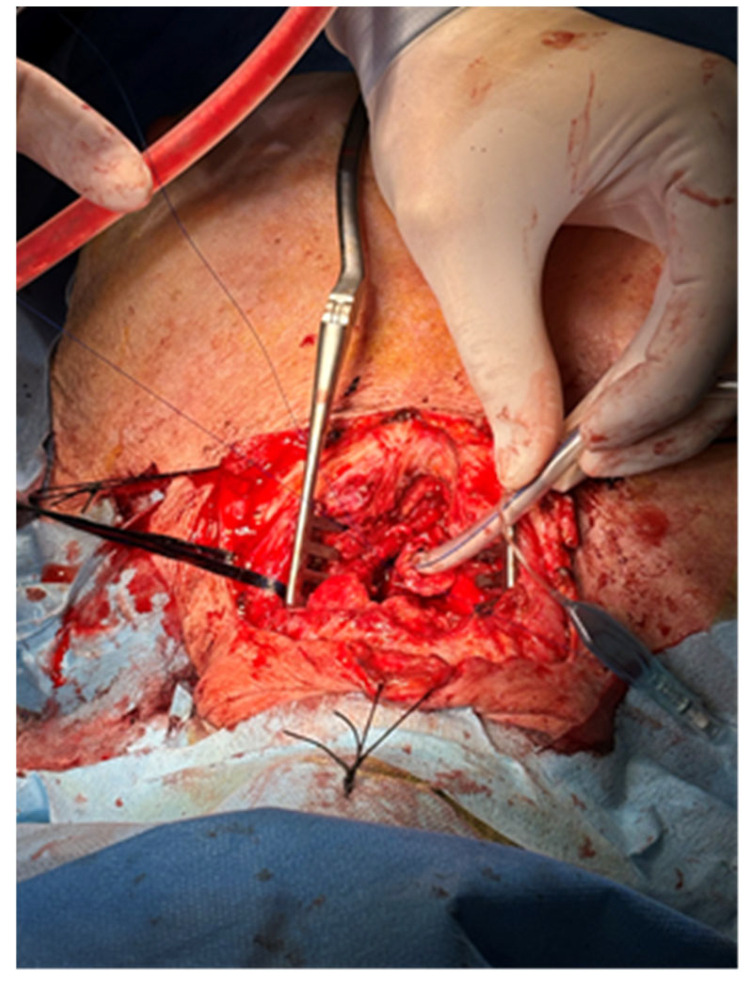
The trachea is repaired just around the endotracheal tube used for intermittent ventilation.

We re-opened the cervicotomy and the anterior wall of the trachea (Figure 1). The oesophagus was repaired with two layers of 3-0 non-absorbable polypropylene running suture, and the same type of suture was used for the posterior tracheal wall. At the end of the procedure, a tracheotomic cannula was positioned to maintain airway patency and to ensure safe ventilation (Figure 2). On the following day, a gastrostomy in the left hypocondrium was performed by general surgeons to prevent reflux.

**Figure 3:**
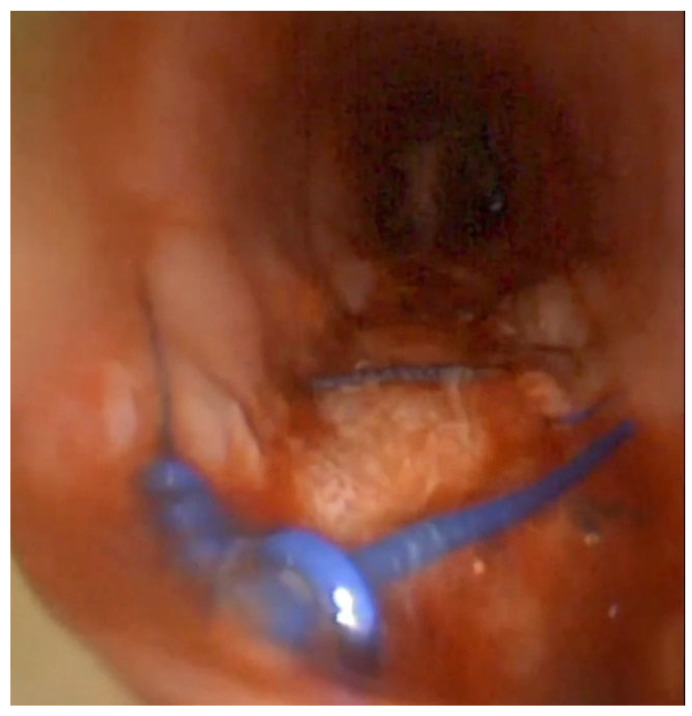
The biological prosthesis sutured to the healthy trachea.

Three days after the second surgery, a significant limitation to ventilation reoccurred in the ICU, and bronchoscopy confirmed complete dehiscence of the sutures. A muscle-sparing posterolateral thoracotomy was performed, exposing the oesophagus and the trachea. This revealed that tissues were highly compromised, with ischemic areas being present, especially on the tracheal wall. We then performed an oesophagectomy and repaired the membranous wall of the trachea with a biological prosthesis sutured with single stitches to the apparently healthy tracheal tissue (Figure 3). We used a biologic prosthetic tissue, specifically, a decellularised porcine dermis material which has proven effective in surgical applications; indeed, such prosthetic materials are part of the standard equipment at our institute. We used the homolateral latissimus dorsi as a flap to protect the prosthesis at this level. We then performed a redo cervicotomy, and a cervical oesophagostomy was created. A right sternocleidomastoid muscle flap was positioned to protect the tracheal prosthesis, and a tracheotomic cannula was positioned. Unfortunately, the patient died four days after the third reintervention due to several septic complications which occurred during the very complex post-surgical management.

## 4. Discussion

Various classification systems have been proposed to standardise the description and management of tracheal lacerations, aiming to improve communication among clinicians and guide therapeutic decision-making. Among these, one of the most widely accepted is that introduced by Cardillo and colleagues [8], which stratifies iatrogenic tracheobronchial lesions into four types based on the depth and extent of the injury. This classification not only guides diagnostic and therapeutic approaches, but also correlates with prognostic outcomes and the likelihood of requiring surgical intervention. Minor, partial-thickness tears involving the mucosa or submucosa (Level I–II) may often be managed conservatively under close bronchoscopic monitoring, whereas full-thickness or complex lesions (Level III–IV) typically necessitate surgical repair, particularly when the oesophagus or carina is involved or when they are accompanied by extensive air leaks, mediastinal emphysema, or respiratory compromise

Cervical tracheal lesions are effectively managed through a cervicotomy incision, which provides excellent exposure of the upper trachea and proximal main bronchi [8]. As already stated, at our centre a hybrid surgical approach is usually employed; this ensures precise suture placement and luminal alignment [7]. The best chance of successful repair of tracheal injuries occurs when all devitalised tissue is removed and primary closure without tension is performed as the initial procedure. The two factors that have the greatest bearing on successful repair are the tension on the suture line and the vascular supply to the wound edges [9,10].

In contrast, complex lacerations extending into the distal bronchi and involving surrounding structures such as the oesophagus generally require a thoracic approach. A right posterolateral thoracotomy through the fourth intercostal space offers optimal exposure for lower tracheal and right main bronchial injuries and for oesophageal involvement, whereas a left thoracotomy may be preferred when the left main bronchus is affected [11]. Often, when recurrences happen and the quality of tissues is compromised, a combined approach, such as cervicotomy with partial sternotomy or thoracotomy, may be required to achieve adequate debridement, primary repair, and correct drainage positioning.

In highly selected patients, when the pars membranacea is extensively damaged, ischaemic, or of corrupted quality, a biologic prosthetic replacement (such as an acellular dermal matrix or pericardial patches) may be considered to restore structural integrity, minimise the risk of dehiscence, and prevent airway collapse. These techniques, although rarely indicated, represent valuable salvage options when conventional repair is not feasible [12].

In extremely severe or relapsing cases, such as the one reported in this study, where multiple repair attempts have failed and tissue viability is compromised, demolitive surgery may represent the only remaining therapeutic option. However, such procedures are associated with high morbidity and significant mortality, and they are often correlated with postoperative complications which may ultimately result in patient demise [13,14].

Therefore, the selection of surgical access and technique should be individualised, according to the site and severity of the lesion, the presence of associated injuries, and the overall clinical condition of the patient.

The final goal should remain to achieve adequate exposure for precise reconstruction while minimising further airway trauma, ensuring a tension-free repair, and promoting optimal healing conditions.

## 5. Conclusions

In conclusion, tracheal lacerations are rare but highly lethal injuries of the airway, with no unanimous consensus on their best management. It is believed that an increasing number of patients with tracheal injuries will be seen in hospital as a consequence of improvements in prehospital medical care and the increased use of percutaneous tracheostomy insertions and large-airway intervention techniques.

Whenever surgical intervention is indicated, patients should be referred to specialised centres to ensure access to the full range of therapeutic options. The surgical procedure has to be performed quickly after a correct evaluation with both CT scan and endoscopic control that can confirm the tracheal laceration. An early diagnosis is necessary: the physician has to recognise all the different ways of presentation of tracheal injury which are summarised above; confirmation with bronchoscopy is also mandatory [15].

The present study illustrates a real-life experience in a high-volume tertiary centre, reflecting the complexity and heterogeneity of these cases.

From a surgical point of view, the majority of lacerations may be safely addressed by means of cervicotomic access with or without a hybrid procedure, as used in selected cases reported here, which involves use of a magnifying scope to additionally manage distal lacerations in the thoracic trachea. When the quality of tissues is compromised or when dehiscences occur, a prosthetic material can be used to reinforce the suture.

In the worst scenarios of severe tracheoesophageal injury, when primary repair is technically unfeasible or when there is extensive tissue devitalisation, oesophagectomy may be necessary to achieve adequate source control and facilitate subsequent reconstruction.

## Figures and Tables

**Table 1:** Classification of lacerations by aetiology, extension, and anatomical location. ETI—endotracheal intubation.

	Aetiology			Extension			Location			Cardillo Classification	
	ETI	Percutaneous Tracheostomy	Other	<2 cm	2–4 cm	>4 cm	Cervical Trachea	Thoracic Trachea	Carina-Bronchus	III	IV
**Patients**	13	4	2	5	4	10	10	5	4	13	6

## Data Availability

The original contributions presented in this study are included in the article. Further inquiries can be directed to the corresponding author.
